# A Chronically Implantable Bidirectional Neural Interface for Non-human Primates

**DOI:** 10.3389/fnins.2017.00514

**Published:** 2017-09-15

**Authors:** Misako Komatsu, Eriko Sugano, Hiroshi Tomita, Naotaka Fujii

**Affiliations:** ^1^Ichinohe Group, Laboratory for Molecular Analysis of Higher Brain Function, RIKEN Brain Science Institute Saitama, Japan; ^2^Department of Chemistry and Biological Sciences, Iwate University Iwate, Japan; ^3^Laboratory for Adaptive Intelligence, RIKEN Brain Science Institute Saitama, Japan

**Keywords:** electrocorticography (ECoG), common marmoset, monkey, optogenetics, brain–machine interface (BMI)

## Abstract

Optogenetics has potential applications in the study of epilepsy and neuroprostheses, and for studies on neural circuit dynamics. However, to achieve translation to clinical usage, optogenetic interfaces that are capable of chronic stimulation and monitoring with minimal brain trauma are required. We aimed to develop a chronically implantable device for photostimulation of the brain of non-human primates. We used a micro-light-emitting diode (LED) array with a flexible polyimide film. The array was combined with a whole-cortex electrocorticographic (ECoG) electrode array for simultaneous photostimulation and recording. Channelrhodopsin-2 (ChR2) was virally transduced into the cerebral cortex of common marmosets, and then the device was epidurally implanted into their brains. We recorded the neural activity during photostimulation of the awake monkeys for 4 months. The neural responses gradually increased after the virus injection for ~8 weeks and remained constant for another 8 weeks. The micro-LED and ECoG arrays allowed semi-invasive simultaneous stimulation and recording during long-term implantation in the brains of non-human primates. The development of this device represents substantial progress in the field of optogenetic applications.

## Introduction

Optogenetic approaches enable the control of neural activity using light-sensitive receptors, which are genetically introduced to neurons. This technology provides many distinct advantages, including millisecond temporal precision (Han and Boyden, 2007; Boyden, 2015), cell-type specificity (Han et al., 2009; Nathanson et al., 2009), and elimination of electrical artifacts (Zhang et al., 2007). Especially, electrical artifacts cannot be avoided from widely-used technologies of neural stimulation such as electrical or electromagnetic techniques. Therefore, optogenetic approaches have a clear advantage over electrical and electromagnetic stimulation in animal studies of clinical applications, as well as for investigating neural circuit dynamics.

To achieve translation to clinical usage, optogenetic interfaces must be capable of chronic stimulation and recording with minimal brain trauma. Most current systems for combined stimulation and recording rely on the use of “optrodes,” which are penetrating electrodes combined with optical fibers (Gradinaru et al., 2007). While optrodes can be moved daily, repeated penetration will damage tissue and degrade local circuits (Gerits et al., 2012). To allow stable monitoring and stimulation at many sites, some recent studies have used a micro-electrocorticographic (μECoG) array combined with micro- light-emitting diodes (LEDs) (Kwon et al., 2013), optical fiber coupled with laser (Ledochowitsch et al., 2015), and transparent silicone artificial dura (Richner et al., 2014; Pashaie et al., 2015; Yazdan-Shahmorad et al., 2016). Whereas, the μECoG array allows for long-term recordings, photostimulations require access routes to the brain and make those devices still invasive.

In this study, we developed a novel, chronically implantable and bidirectional neural interface, which consists of a multi-channel LED and whole-cortical ECoG array. This device enables long-term chronic photostimulation, and simultaneously monitors cortical responses to the stimulation, without any damage to brain tissue and dura mater. The opsin channelrhodopsin-2 (ChR2) was virally transduced in the cerebral cortex of marmosets followed by epidural implantation of the device, which minimizes brain tissue damage. ECoG electrodes were placed on the entire right hemisphere and blue-light LED chips on the right frontal and parietal lobes, and ECoG potentials were monitored during photostimulations for 3 months post-transduction. Our study may thus provide insights into the potential applications of optogenetics, particularly with reference to the effects of photostimulation.

## Methods

This study was carried out in accordance with the recommendations of the National Institutes of Health Guidelines for the Care and Use of Laboratory Animals. The protocol was approved by the RIKEN Ethical Committee (No. H26-2-202). All surgical procedures were performed under anesthesia, and all efforts were made to minimize the number of animals used, and their discomfort.

### Chronically implantable photostimulation device

#### Fabrication of micro-LED array

**Figure 1:**
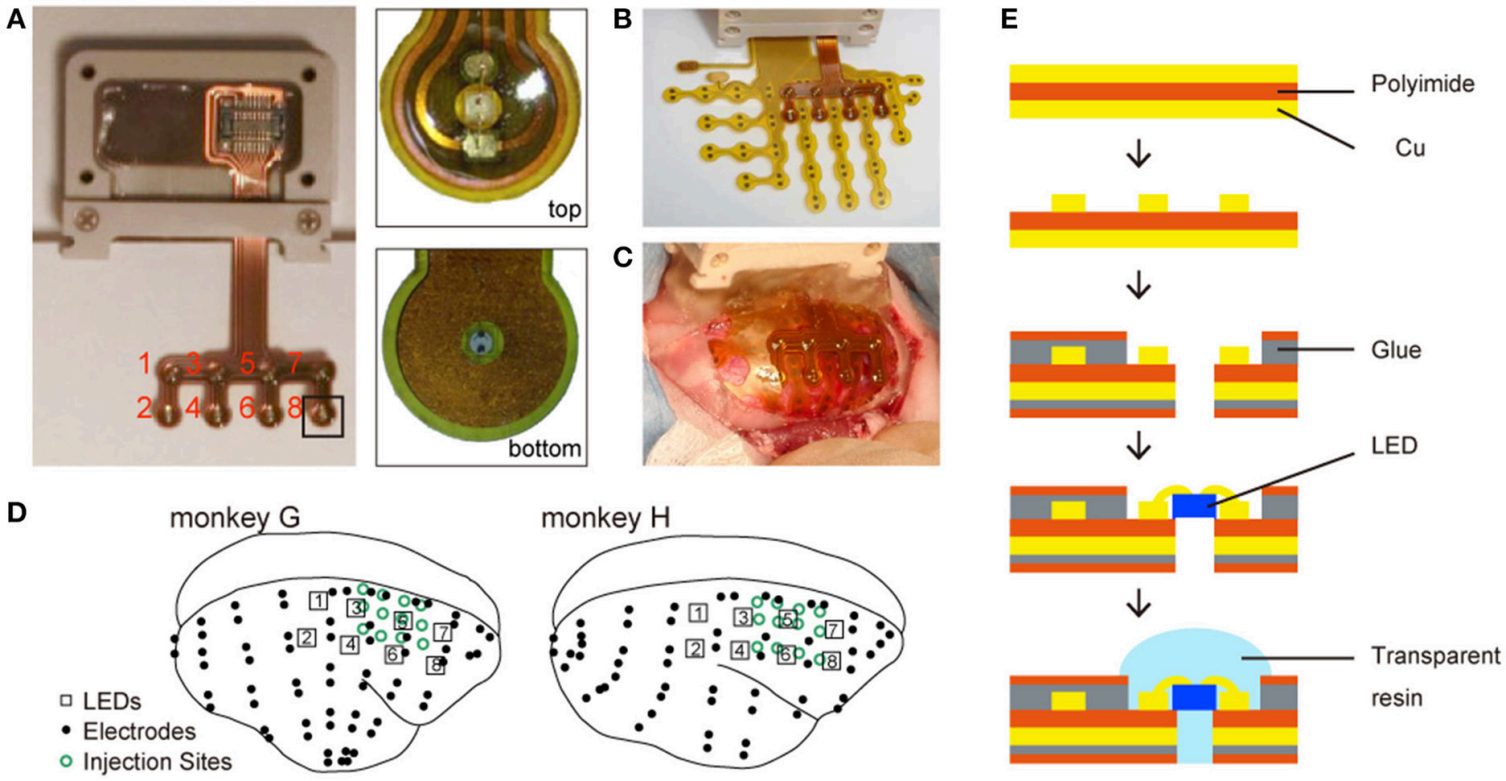
Chronically implantable photostimulation device. **(A)** Left: an overview of the micro-LED array; right: magnified images from the experimenter's (top) and animal's (bottom) perspective. The red numbers indicate the number of each LED. **(B)** The LED and ECoG arrays. **(C)** An example of electrode implantation. **(D)** Locations of LEDs (numbered squares), electrodes (black dots), and virus injection sites (open green circles). **(E)** Fabrication of the LED array.

The chronically implantable micro-LED array (Figure 1) was manufactured by Cir-Tech Inc. (Shizuoka, Japan) for optical stimulations. The base of the array, made of a thin, flexible, circuit board material, consisted of a layer of polyimide film sandwiched between two copper layers. The copper layer on one side was dry etched for wiring the LED to the connector, and the copper layer on the other side was shaped for an electric shield. The copper layers were insulated with a polyimide film attached with glue. Then, holes were made by laser drilling at the desired locations and LEDs (ES-CEBHV13; EPISTAR Corp., Hsinchu, Taiwan) were installed in the holes. Finally, all the LEDs were sealed with transparent resin.

The array consisted of eight LEDs soldered to a narrow-pitch connector. The connector enclosure was made of PEEK resin. The distance between two LEDs was 4 mm. Each LED emitted blue light (470 nm wavelength) and was 330 × 330 μm in size.

#### Implement with ECoG electrodes

Customized multichannel ECoG electrode-arrays (Cir-Tech Inc., Shizuoka, Japan) (Komatsu et al., 2015) were used for neural recordings (Figure 1D). Each electrode contact was 0.6 mm in diameter and had an inter-electrode distance of 1.4 mm in a bipolar pair.

### Preparation of ChR2-venus-expressing animals

#### Subjects

We used two adult male common marmosets (*Callithrix jacchus*) that weighed 320–380 g. The monkeys were injected with adeno-associated virus (AAV)-ChR2 and then implanted with the LED array and ECoG electrode array in the epidural space on the same day. All surgical procedures were performed under general anesthesia.

#### Vector construction

The construction of the vector expressing ChR2 and the preparation of the vector for injection has been described in detail (Sugano et al., 2005; Tomita et al., 2007). The N-terminal fragment (residues 1–315; GenBank Accession No. AF461397) of the ChR2 gene was fused to a fluorescent protein, Venus, in frame at the end of the ChR2 coding fragment. Then ChR2-Venus (ChR2V) was introduced into the EcoRI and HindIII sites of the 6P1 plasmid. The synapsin promoter was exchanged for a hybrid cytomegalovirus (CMV) enhancer/chicken β-actin promoter (CAG) (Niwa et al., 1991). Site-directed mutagenesis of tyrosine-to-phenylalanine on AAV8 capsids was performed according to Petrs-Silva et al. (2009). AAV8 mutated virus vector (Petrs-Silva et al., 2009), AAV8M-ChR2V, was purified by the single-step column purification method of Auricchio et al. (Auricchio et al., 2001; Sugano et al., 2005).

#### AAV vector injection

The animals were initially sedated with butorphanol (0.2 mg/kg; intramuscular [i.m.] administration), and surgical anesthesia was achieved with ketamine (30 mg/kg; i.m.) and medetomidine (350 μg/kg; i.m.). The animals were then positioned in a stereotaxic frame (Narishige, Japan) and placed on a heating pad during surgery. Vital signs were monitored throughout surgery. Virus injection and implantation of the electrode array involved the removal of a bone flap (~2 cm along the anterior-posterior axis and ~1 cm along the mediolateral axis) over the parietal cortex.

For the viral injection, we used a handmade injection device. A 9-cm length of a 32G SUS hypodermic needle (15° angle), which was encased in a 23G SUS tube to ensure strength, was connected to a 2 μl Hamilton syringe through a 23G PE tube. This device allowed us to inject the virus through the dura mater and to minimize suffering. The device was filled with mineral oil, and we discarded the oil to scale mark zero on the Hamilton syringe. Then, 2 μl of viral solution (4.17E+12 capsides/ml) was withdrawn and injected at a rate of 1 μl/min to a depth of 2 mm below the cortical surface. After each injection, the needle was maintained in place for an additional 1 min, and then slowly withdrawn. We repeated this injection procedure at 12 sites with 2 mm between-site separation, at A8–A14 and L3–L7 in each animal.

#### Histological validation

Monkeys G and H were sacrificed 20 and 14 weeks after injection, respectively. For monkey G, the brain was removed and fixed with 4% paraformaldehyde in 0.1 M phosphate buffer. Part of the brain was embedded in optimum cutting temperature (OCT) compound (Sakura, Tokyo, Japan) after immersion in 30% sucrose solution with phosphate-buffered saline (PBS). Sections of 500-μm thickness were produced and mounted on slides. Slides of the sections were covered with medium (Vectashield; Vector Laboratories, Burlingame, CA). Venus fluorescence was visualized under a fluorescence microscope (Axiovert 40; Carl Zeiss, Oberkochen, Germany) and the images were stored. Monkey H was perfused transcardially with 0.9% NaCl, followed by fixation with 4% paraformaldehyde in 0.1 M phosphate buffer. The brain was postfixed in 4% paraformaldehyde for 12 h and sunk in 30% sucrose in PBS. Then we cut 50-μm-thick coronal sections. Every continuous three sections were grouped together. Each section of the group was immunohistochemically stained for NeuN, myelin, or Nissl substrates, respectively. The latter two section series were used to identify brain areas. The first sections were incubated with the primary antibody for neuron-specific nuclear protein (NeuN; mouse monoclonal, MAB377; Millipore, Billerica, MA). We used AlexaFluor 568 (1:100; Invitrogen A11036) as secondary antibodies. Venus fluorescence was visualized under a fluorescence microscope and the images were stored. For areal demarcation, the sections of the second series were stained for myelin (Pistorio et al., 2006), and those of the third series were stained for Nissl substrate with thionin.

### Performance examination in awake animals

#### Implantation of LED and ECoG array

**Figure 2:**
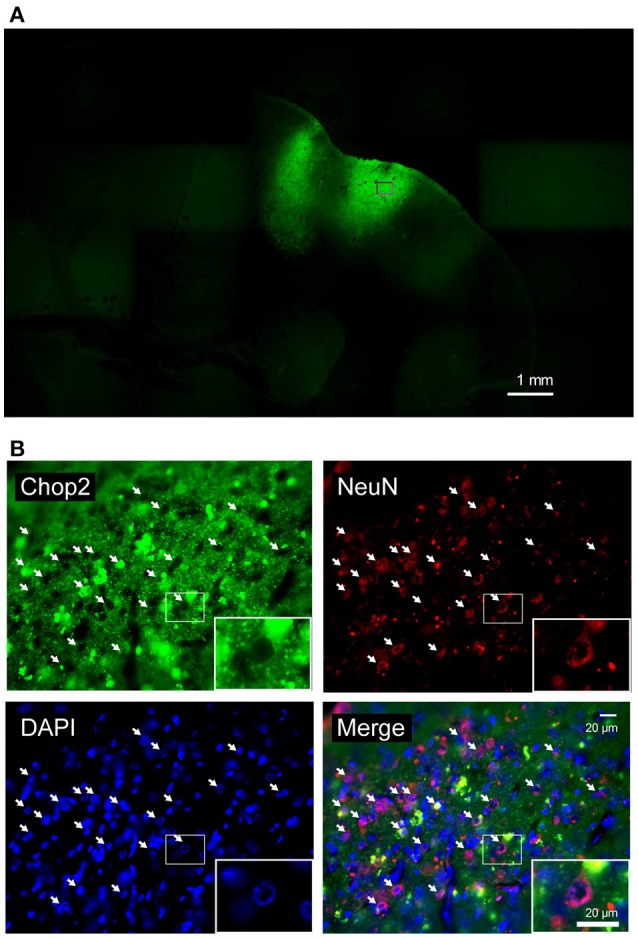
Fluorescent images of marmoset's brain expressing ChR2V. **(A)** Coronal slice through the right hemisphere of monkey G. **(B)** Magnified views of an injected area (indicated by rectangle). ChR2V was expressed in the membrane of the cells, whereas NeuN expression was observed in the nuclei and cytoplasm of neuronal cells. Arrows point to ChR2V expression on the membrane of cell bodies of neurons.

We implanted eight LEDs and 64 (monkey G) and 58 (monkey H) electrodes in the epidural space of the right hemisphere. The electrode array covered the frontal, parietal, temporal, and occipital lobes. Following virus injection, part of the ECoG array was implanted in the epidural space. After positioning the array, connectors were attached to the skull using dental acrylic and PEEK screws (size 1.4 × 2.5 mm). The reference electrodes were placed in the epidural space and the ground electrodes in the episkull space. The anti-inflammatory corticosteroid dexamethasone (2.0 mg/kg; i.m.) was administered after surgery to prevent brain swelling. The animals were administered antibiotics and analgesics daily for 5 days after surgery. Following the animals' recovery, the position of each electrode in the arrays was identified based on computer tomography, and then co-registered to pre-acquired T1-weighted anatomical magnetic resonance images using MRIcron software (http://www.mricro.com). In both monkeys, the electrode array covered the frontal, parietal, occipital, and temporal cortices and the LED array covered the parietal to frontal cortices (Figure 2).

#### Photo-stimulation

Photostimuli were delivered using the micro-LED array via a Universal Serial Bus (USB) input/output device (USB1308FS; Computing Measurements Inc., Norton, MA) or multi-channel stimulator (MCS101; Unique Medical, Japan). The USB1308FS delivered analog voltage to the array and was used for random illumination of LEDs. The MCS101 delivered digital current stimuli to the array and was used for simultaneous multichannel stimulation. Both stimulus presentations were controlled by MATLAB (MathWorks Inc., Natick, MA) using the Psychophysics Toolbox extensions (Brainard, 1997; Pelli, 1997).

Every week, for 1–12 and 1–8 weeks after the virus injections of monkey G and H, respectively, we applied 3.5 V to each LED for 200 ms to monitor the development of neural responses to photostimuli. In each session, eight LEDs were pseudo-randomly illuminated and 50 stimulation trials were performed for every LED. Inter stimulus intervals were fixed at 2 s. Eight weeks after the injection of monkey G, the effects of light intensity were explored at various voltages (1.5, 2.0, 2.2, 2.4, 2.6, 2.8, 3.0, and 3.5 V) with the same settings. Furthermore, by using the MCS101 device, we examined the effect of light pulses of various durations (5, 10, and 20 ms) and the effect of trains of those pulses at high- and low-frequencies (10 pulses delivered at 50 and 10% of duty ratio).

#### ECoG recordings and preprocessing

ECoG recordings were obtained with monkeys awake and resting. In each recording session, the monkey sat in a primate chair in a dimly lit room. The length of each recording was ~15 min.

ECoG signals and stimulus onsets were recorded at a sampling rate of 1 kHz, using a Cerebus Data Acquisition System (Blackrock Microsystems, Salt Lake City, UT). In the signal preprocessing, signals were re-referenced using a common median reference (CMR) montage. We segmented datasets from −100 to 2,000 ms relative to the onsets of the photostimuli. To remove the segments that contained outliers, we calculated a standard deviation (*SD*_*i*_, i = 1–50) for each segment and for all segments (*SD*_all_) and rejected segments with an *SD*_*i*_ above 3 *SD*_all_. After outlier rejection, we applied a baseline correction by subtracting the mean of the 100 ms period before the stimulus onset. The event-related potentials (ERPs) were then calculated for each channel. To distinguish spontaneous signals in the baseline period and responses to photostimuli, we conducted Wilcoxon rank sum tests for each LED, each channel at baseline and each time point in the 0–2,000 ms after stimulus onset. All *p*-values from each monkey and each recording session were adjusted for false discovery rate (FDR) and significance level was 0.001 unless otherwise specified. Parts of the dataset are shared on the public server Neurotycho.org (http://neurotycho.org/) (Nagasaka et al., 2011).

## Results

### ChR2V expression in the brain of marmosets

We used standard histological techniques to investigate virus infection and opsin expression in the marmosets' brains with the nuclear marker 4',6-diamidino-2-phenylindole (DAPI) and the neuron-specific nuclear marker NeuN (Figure 2). ChR2V was expressed in the membrane of the cells, whereas NeuN expression was observed in the nuclei and cytoplasm of neuronal cells. Merged images showed that the ChR2V was expressed in the membrane of the NeuN-labeled cells, which meant that the expression of ChR2 successfully occurred in neurons. Furthermore, cell nuclei of infected cells appeared to be intact and the morphology of infected neurons appeared normal 2–3 months after injections.

### Neural responses to photostimulation

**Figure 3:**
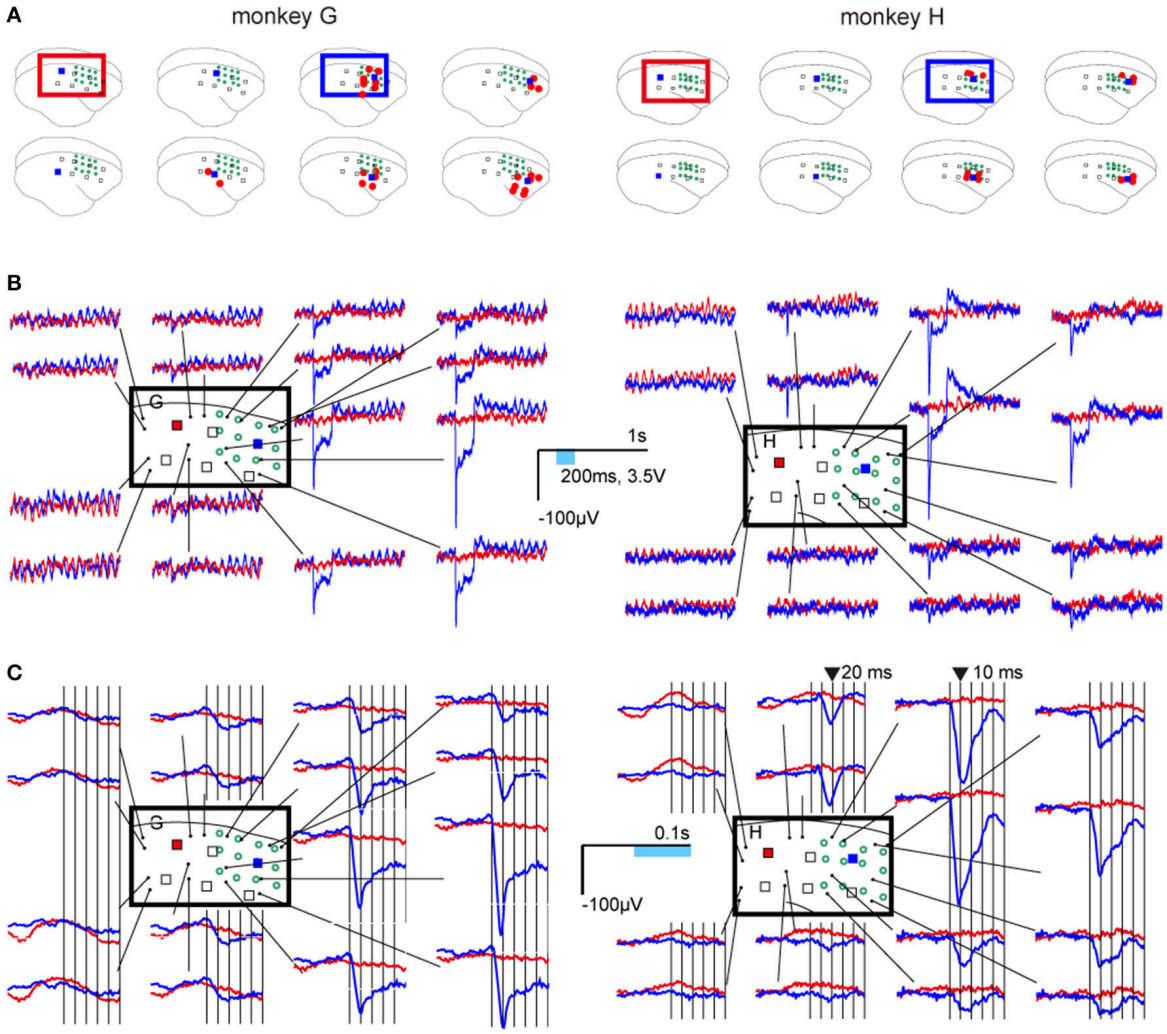
Neural responses to photostimulations. **(A)** Locations of ON-LEDs (blue squares) and electrodes showing statistically significant changes in neural activity (red dots; adjusted *p* < 0.001). The open green circles indicate virus injection sites. **(B)** Examples of event-related potentials (ERPs). Blue and red lines show the average waveforms of responses to LED5 in the injection area (blue square) and to LED1 in an area that was not injected (red square), respectively. **(C)** Temporally magnified views of the ERPs. The ERPs 50 ms before and after stimulus onset are shown for LED5 and LED1. The vertical bars indicate each 10 ms after stimulus onset.

Eight weeks after the injection, significant responses were observed at electrodes adjacent to lighted LEDs (ON-LEDs) in the injected area, and not observed at any electrodes adjacent to ON-LEDs in the areas that were not injected (Figure 3A). In both monkeys, the ERPs of the responses to 200-ms single-pulse stimuli were characterized as follows: (1) high-amplitude and sharp negativity followed the photostimuli onsets, (2) low-amplitude and sustained negativity during stimulation, and (3) after the stimulus offset, overshoot preceding return to baseline (Figure 3B). The amplitudes of these responses strongly depended on the distance between the ON-LED and electrode.

**Figure 4:**
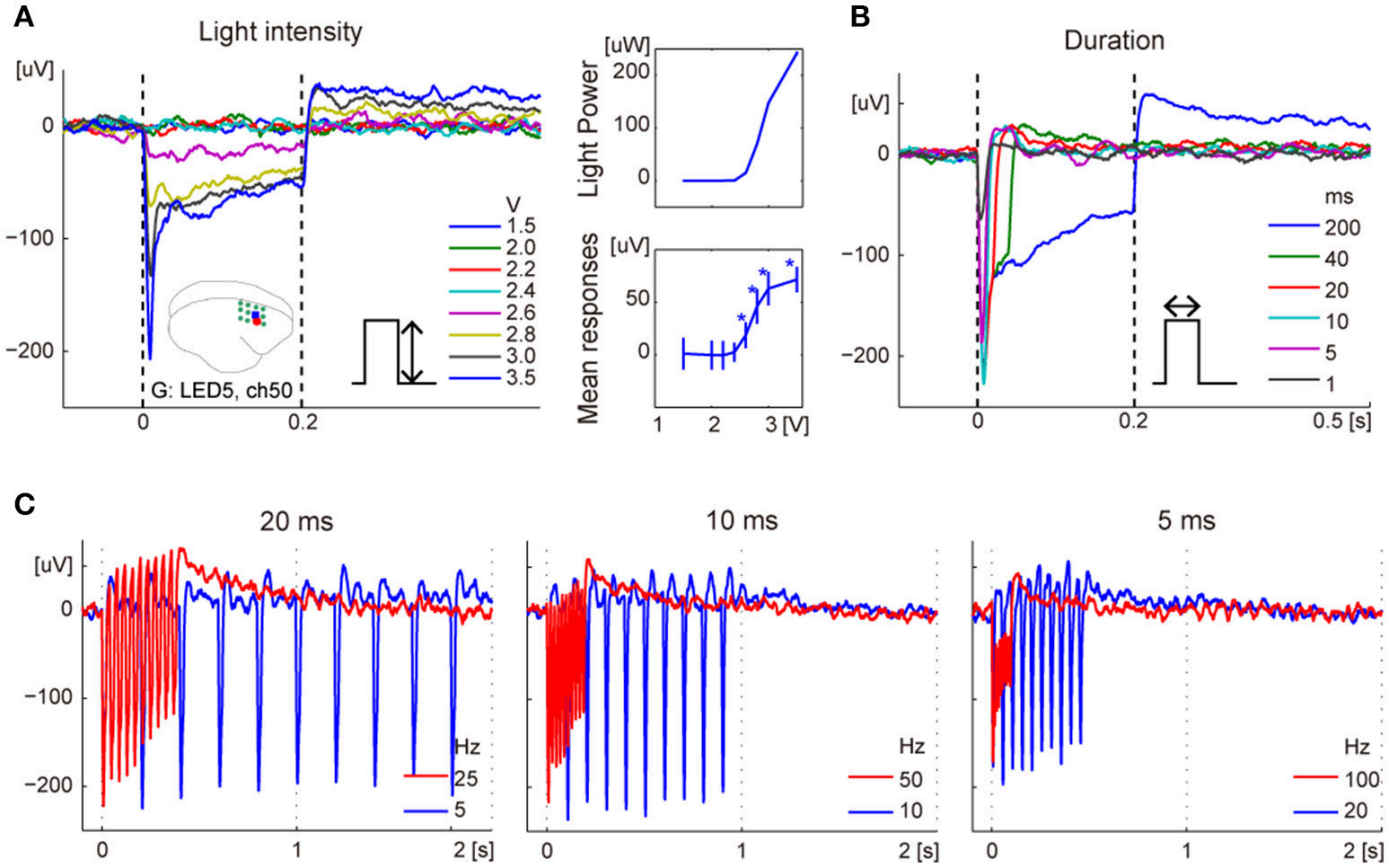
Typical responses to variation of photostimulations. **(A)** Dependency on LED power in ERPs (left) and in amplitudes of mean responses during photostimulations (bottom right). The error bars show confidence intervals. The asterisks indicate significant responses. The top right inset shows light intensity corresponding to voltage applied. The brain icon shows locations of the recording electrode (ch43; red dot) and ON-LED (LED5; blue square). The green circles indicate locations of the virus injection sites. **(B)** Dependency on the duration of photostimulation. **(C)** ERPs for 10-pulse stimulations of 20 ms (left), 10 ms (center), or 5 ms (right) duration at high- and low-frequency. The frequencies were 50 and 10% duty cycle for each stimulus duration.

We examined changes in the responses for various light intensities, pulse durations, and pulse frequencies, 8, 13, and 14 weeks after the injection of monkey G, respectively. Regarding dependency on LED power, the amplitudes of the mean responses during stimulation increased with stimulus intensity, while peak latencies did not change. The spatial distribution of the responses also depended on the light intensity. For example, of the responses induced by LED5, significant responses were observed for stimuli exceeding 2.6 V from the nearest electrode, while a distant electrode showed light induced activity for stimuli exceeding 2.8 V LED power (Figure 4A, Supplementary Figure 1).

Photostimulation of different durations led to similar responses until stimulus offset, and the potentials returned to baseline after their offset (Figure 4B). Furthermore, we examined the responses to 10-pulse photostimulations at 50 and 10% duty ratio, with 5, 10, or 20-ms stimulus duration. For the same duration, low-frequency stimuli showed larger peak values than high-frequency stimuli (Figure 4C).

### Development of responses after injection

**Figure 5:**
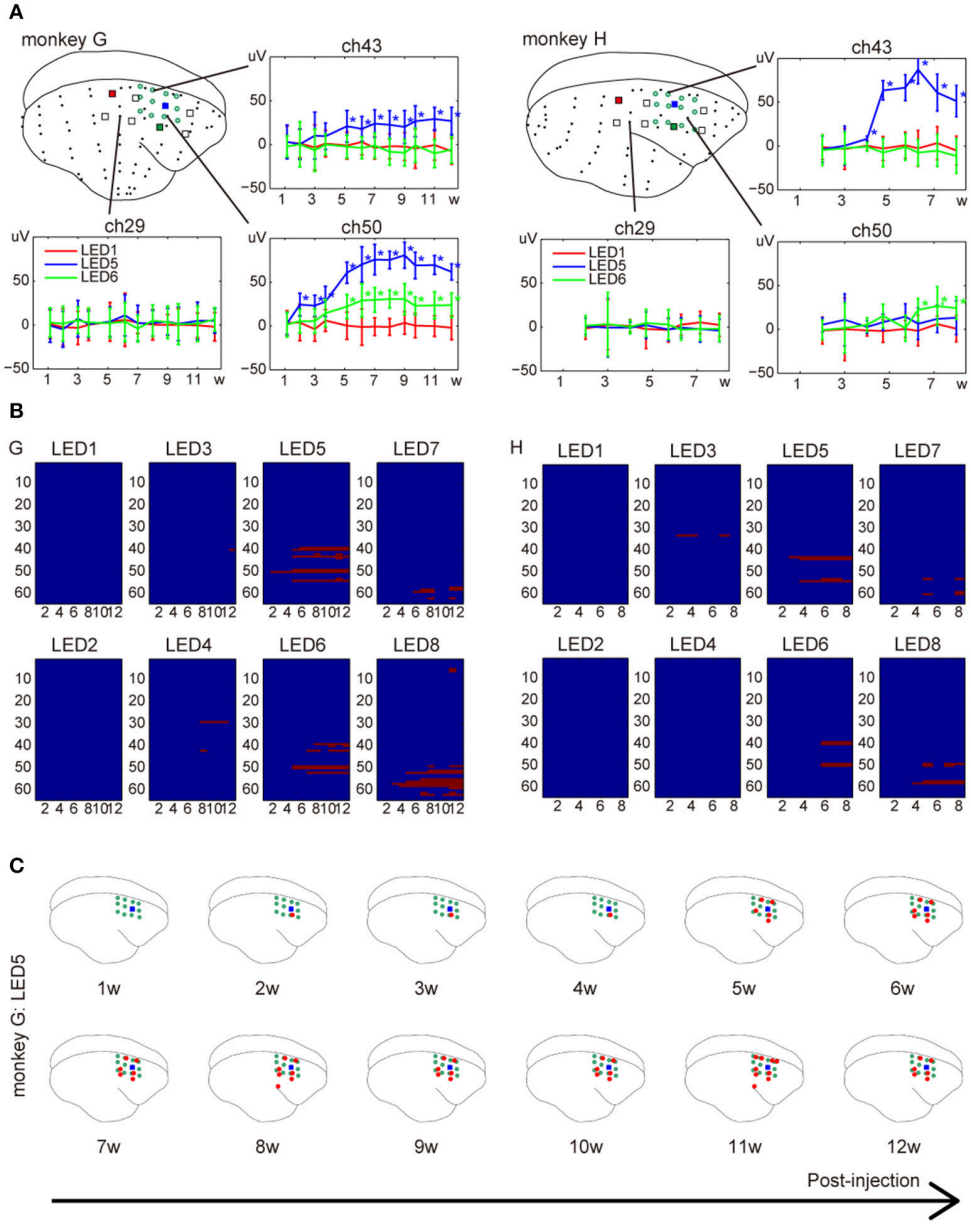
Development of responses over the week following injection of AAV-ChR2V. **(A)** Changes in amplitude. The error bars show confidence intervals. The asterisks indicate significant responses (adjusted *p* < 0.0001). **(B)** Changes in spatial distribution. The x-axis represents weeks and y-axis lists labels of ECoG electrodes. The red pixels indicate electrodes that showed significant responses at the given week after the injection. **(C)** An example of the spatial distribution at different weeks after the injection. Data were acquired from monkey G.

We monitored responses to a 200-ms light pulse for 3 and 2 months after injection in monkey G and H, respectively. Responses to photostimuli in marmosets injected with AAV8-ChR2 were first detected 2 weeks after the injection (Figure 5). Thereafter, amplitudes progressively increased during the 5-week post-injection period. Responses were not detected with the same stimuli near non-injected sites. Furthermore, we observed changes in the number of electrodes activated, as well as in the mean response amplitudes. Responses were first detected at the nearest electrode to an ON-LED. The number of significant electrodes progressively increased until ~6 weeks post-injection.

## Discussion

The present study demonstrated a multi-channel LED array compatible with an array for whole-cortical ECoG, which enables long-term chronic photostimulation and simultaneous monitoring of cortical responses to stimulation in non-human primates. The significant responses were observed at electrodes adjacent to illuminated LEDs (ON-LEDs) in the injection area, and not observed at any electrodes adjacent to ON-LEDs in the non-injected areas (Figure 3). These results indicate that the neural responses to light stimuli were introduced by the transduction of ChR2V, rather than by electrical or photic artifacts. We examined the response properties to different stimulation parameters, such as light intensities, pulse durations, and pulse frequencies. The amplitudes and spatial distributions of the responses depended on stimulus intensity. These are natural consequences of the facts that higher-intensity light activates a larger population of neurons and reaches more distant neurons. Furthermore, the low-frequency stimuli generated larger peak values than high-frequency stimuli. This result might have a physiological origin, such as opsin's off-kinetics, the neuron's refractory period, and/or local circuit dynamics involving excitatory and inhibitory neurons (Osawa et al., 2013; Ledochowitsch et al., 2015). We note that the spatial extent of light induced activity differs from each LED (Figure 5C). This was probably caused by different amounts of ChR2 expressions and/or local circuits around the LED. Thus, in order to apply our device to specific brain functions, researchers need to carefully determine how to transduce opsins and where to locate LEDs.

An important aspect of a chronically implantable device is its functional lifetime. There is considerable evidence that long-term optogenetic stimulation, viral toxicity, and instability of expression over time can lead to decreased photo response within months. In this study, post-injection monitoring revealed response developments in terms of amplitude and spatial distribution, and the stability of our devices in awake marmosets. The histological examinations also revealed that cell nuclei of infected cells appeared intact over 3 months after the injections, despite the cells being repeatedly exposed to photostimulations (Figure 2). These results indicate that our device is capable of chronic monitoring of photostimulation effects; this represents an effective application of optogenetics. Furthermore, Sugano et al. (2011) have reported ChR2V functions stably across 64 weeks in retinas of rats and does not affect retinal histology. Further studies are needed to determine whether these findings are also applicable to the brains of non-human primates.

For clinical applications, validation with non-human primates is inevitable. Most applications of optogenetic methods have been conducted in rodents. Recently, several studies have demonstrated the potential for manipulating neural circuits with optogenetic technologies in primates, namely macaque monkeys (Gerits and Vanduffel, 2013). However, the behavioral effects observed in such studies have been notably less robust than in many of the comparable rodent studies (Gerits and Vanduffel, 2013). Several factors likely contributed to these outcomes, and future studies are required to optimize these methods for primates. In this study, we utilized marmosets as a non-human primate model. Although the marmoset has emerged as a potentially important model in neuroscience, to our knowledge only one optogenetics study has been conducted previously on the marmoset (MacDougall et al., 2016). The brain of the marmoset is entirely lissencephalic. This feature allows direct access to most cortical areas directly beneath the surface of the brain; thus, our device allows for complete manipulation of the entire cortical network. Another advantage of the marmoset is its suitability for gene-editing techniques (Sasaki et al., 2009; Izpisua Belmonte et al., 2015). While opsins expressed in transgenic mice have facilitated optogenetics study (Ting and Feng, 2013), to date, models of transgenic monkeys that express opsins have not been successfully generated. In future, we expect to generate a transgenic opsin-expressing marmoset model; such a model would accelerate our understanding of the mechanisms underlying neural diseases by enabling optogenetic approaches. Within this framework, our whole-cortex monitoring and stimulation device will be a powerful optogenetic tool.

## Conclusion

The micro-LED and ECoG arrays permitted semi-invasive simultaneous recording and stimulation from long-term chronic implantation into the brain of non-human primates. This device represents substantial progress in clinical applications of optogenetics.

## Author contributions

MK and NF designed and conducted the experiments, and analyzed the data for development of the device. ES and HT designed and conducted the viral preparation and histological validation. All authors drafted the manuscript and approved the current version of this paper.

### Conflict of interest statement

The authors declare that the research was conducted in the absence of any commercial or financial relationships that could be construed as a potential conflict of interest.
