# Supplementary material for: A Chronically Implantable Bidirectional Neural Interface for Non-human Primates
Source: Front Neurosci. 2017 Sep 15;11:514. doi: 10.3389/fnins.2017.00514 (PMC5605616; doi:10.3389/fnins.2017.00514)
Supplement: Supplementary file 1 [file Image1.pdf]

Supplementary Material

A chronically implantable bidirectional neural interface for non-human primates

Misako Komatsu[1]*, Eriko Sugano[2], Hiroshi Tomita[2], Naotaka Fujii[3]*

[1]Ichinohe Group, Laboratory for Molecular Analysis of Higher Brain Function, RIKEN Brain Science Institute, Saitama, Japan

[2]Department of Chemistry and Biological Sciences, Iwate University, Iwate, Japan

[3]Laboratory for Adaptive Intelligence, RIKEN Brain Science Institute, Saitama, Japan

*** Correspondence:**
Misako Komatsu
mkomatsu@brain.riken.jp

Naotaka Fujii
na@brain.riken.jp

1 Supplementary Figure

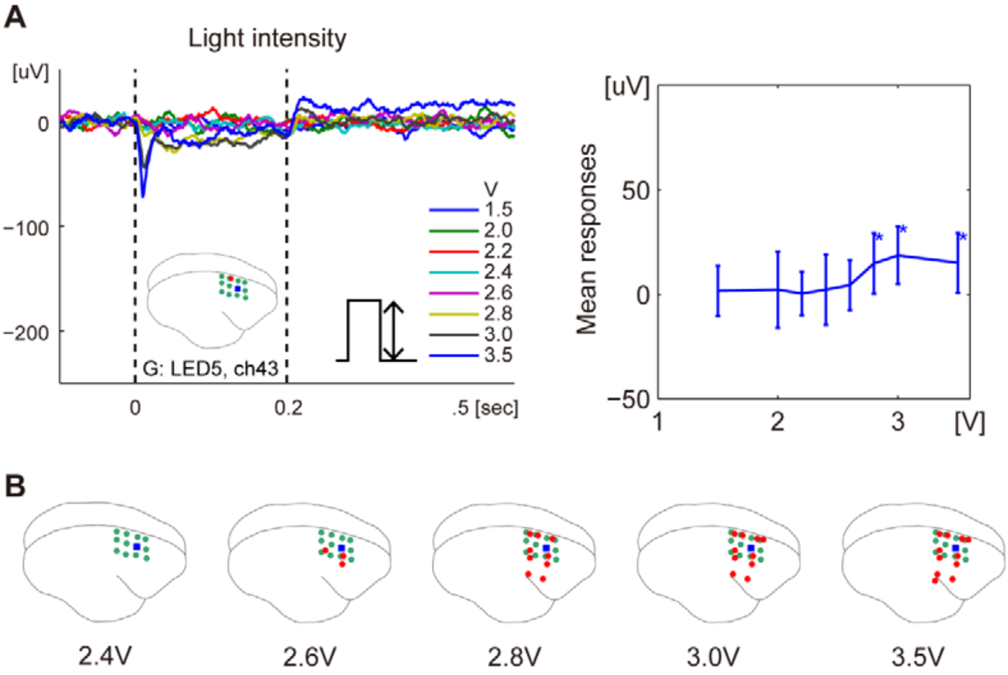

Supplementary Figure 1. Dependency on LED power. (A) Typical responses at a distant electrode (the red dot in the brain icon) in ERPs (left) and in amplitudes of mean responses during photostimulations (right). The error bars show confidence intervals. The asterisks indicate significant responses. (B) Spatial extent of light induced activities. The red dots correspond to the electrodes showing significant changes on neural responses. The blue square and green circles indicate locations of the ON-LED (LED5) and virus injection sites, respectively.
